# Parental Postnatal Depression in the Time of the COVID-19 Pandemic: A Systematic Review of Its Effects on the Parent–Child Relationship and the Child’s Developmental Outcomes

**DOI:** 10.3390/ijerph20032018

**Published:** 2023-01-21

**Authors:** Genova Federica, Tambelli Renata, Eleonora Marzilli

**Affiliations:** 1 Department of Psychology “Renzo Canestrari”, University of Bologna, 40127 Bologna, Italy; 2 Department of Dynamic, Clinical & Health Psychology, Sapienza University of Rome, 00185 Rome, Italy

**Keywords:** perinatal depression, mothers, fathers, early dyadic relationship, early infant development, COVID-19 pandemic

## Abstract

The international literature has shown that maternal and paternal postnatal depression (PND) is one of the most common mental illnesses in the perinatal period, with significant consequences for parent–infant relationships and infant development. The COVID-19 pandemic has increased the rates of prevalence of PND, exacerbating the mental health risk for new families. This systematic review aims to examine the effect of maternal and paternal PND on parent–infant relationships and children’s development in the first 36 months after childbirth during the COVID-19 outbreak. Eligible studies were identified using the following databases: Medline, CINAHL, SCOPUS, PsycINFO, PsycARTICLES, ScienceDirect, and Web of Science. Of the 1252 studies considered, 10 studies met the inclusion criteria. Results showed that maternal PND significantly affected the quality of the early mother–infant relationship and the infant’s motor, self-regulation, and socio-emotional development. In addition, the detrimental impact of maternal PND on the quality of early mother–infant relationships seems to become stronger as COVID-19 concerns increase. No studies included fathers. These findings strengthened the importance of planning targeted prevention and treatment strategies to prevent PND and its short- and long-term consequences, especially in the case of stressful and traumatic events. They also suggested the urgent need for further exploration of fathers.

## 1. Introduction

The novel coronavirus disease 2019 (COVID-19) emerged in Wuhan, China at the end of 2019 and was declared a global pandemic on 11 March 2020 by the World Health Organization (WHO) [1]. To reduce its rapid spread around the world, governments imposed restrictive measures (e.g., lockdowns, quarantines, and social distancing) that have significantly affected the mental health of the general population [2,3]. In this scenario, maternal mental health is particularly important to consider, especially during the perinatal period, due to the increased risk of the onset of psychopathological problems that this phase entails [4]. The perinatal period encompasses the time frame from pregnancy to the first 18–24 months post-birth of the child [5] and represents a delicate stage in which significant psychological, physiological, and social changes occur in parents’ lives [6]. Some women may show difficulties in adapting to these new configurations [7], manifesting an increased risk of developing emotional disorders [4], especially in the presence of recent stressful living conditions [8,9]. Similarly, for fathers, the birth of a child entails major transformations related to the necessity of adapting to the child’s new rhythms and needs, and having to redefine one’s relationships and self-identity, which may be associated with increased levels of stress, anxiety, and depression [10]. In this context, the COVID-19 outbreak and its related restrictions, resulting in a worldwide traumatic experience [11], posed an additional risk to the mental health of postpartum women and men who were shown to be one of the high-risk group populations for negative effects of the pandemic on psychological well-being [12,13,14]. Even before the advent of the COVID-19 pandemic, studies conducted during previous natural disasters had shown higher rates of psychiatric disorders among perinatal women with respect to the general population [15]. Specifically, during the COVID-19 pandemic, international studies have suggested the presence of two main stressors for this group during the perinatal period: stress resulting from feelings of uncertainty and unpreparedness for birth, and stress resulting from fears about their unborn children’s health and their own health [16]. Containment measures have also led to increased concerns about family finances [17,18], reduced social support and higher feelings of isolation [19,20], a dramatic increase in domestic violence [21], and difficulties in accessing professional services [22]. Furthermore, in many countries around the world, fathers have been prohibited from being present during prenatal visits [23] and, in some cases, even during the birth of their infants [24]. As a consequence, the birth experience, once a “couple event,” has for many mothers and fathers been experienced as a “single event,” negatively conditioning the affective experience of the new family [25]. Moreover, during the postpartum period, the mother’s stress, when accompanied by high levels of the father’s stress, can pose an additional risk to the child’s development [26]. Overall, COVID-19 has further complicated an already richly complex phase of mental health for new parents [4], increasing the risk of the onset of mental health illness after childbirth [27], especially depressive symptoms.

Postnatal depression (PND), also known as postpartum depression (PPD), is a mood disorder defined as major or sub-clinical depression typically affecting women within one year of delivery [28,29]. Generally, PND symptoms are accompanied by other psychopathological symptoms, including irritability, anxiety, sleep difficulties, and low appetite [30]. Feeling overwhelmed and obsessing about the child’s health and nutrition have also been reported [31]. Moreover, some women with PND may experience self-harm ideation and worry about causing harm to their children [32]. Pre-pandemic epidemiological studies have shown that PND is the most common postpartum disorder among women [33], reporting a worldwide incidence ranging from 6.5% to 25.8% until 2019 [34,35]. During the COVID-19 pandemic, two recent meta-analyses [36,37] have reported prevalence rates of PND between 32.6% and 34%, evidencing a dramatic increase compared to the incidence found during the pre-pandemic period.

Interestingly, accumulating research has shown that fathers may also suffer from PND [38,39], especially in developing countries [40]. Epidemiological studies conducted before the pandemic reported prevalence rates of paternal PND between 2% and 25% [41,42]. The few studies exploring paternal PND during the COVID-19 pandemic have confirmed the clinical relevance of this phenomenon, reporting prevalence rates from 13.8% to 21.2% [43,44]. Moreover, this incidence tends to increase with the presence of maternal PND [45], supporting the importance of focusing on both maternal and paternal mental health in studying the psychopathological effects of the COVID-19 pandemic on families during the perinatal period. 

However, beyond the negative effect of PND on parents’ mental health, studies conducted before the pandemic evidenced the effect of significant short- and long-term consequences on children’s health [46,47]. Moreover, prior research has suggested that maternal PND increases the risk for the development of children’s social, emotional, and behavioral problems [48,49], which might persist over time [50,51,52]. Cognitive, language, and motor developmental problems among offspring of mothers suffering from PND have also been reported [46,53]. Regarding fathers, a recent meta-analysis by Low et al. [47] has shown that paternal PND was prospectively associated with children’s internalizing and externalizing problems. 

A possible explanation of the negative impact of PND on children’s developmental outcomes is that the neonatal period represents one of the critical developmental stages with the greatest neuronal plasticity [54], in which a gene x environment interaction could exert its risk effects through epigenetic modifications [55]. After birth, among the environmental influences resulting from parental care (e.g., sensory and physical stimulation and nurturing), the quality of the mother–infant and the father–infant relationship has been proposed as a crucial risk factor for child development [56,57]. In this context, the life-course perspective [58,59] offers a valid model for understanding the intergenerational transmission of psychopathological difficulties from parents to children, considering individual development as the result of a complex pathway of risk and protective factors during the life course, especially within the family context. Specifically, in line with the notion of “linked lives” [60], parents and children represent a latent network of support in which the life course of each family member mutually influences the psychological well-being of members of the other generations [61]. Coherently, stressful life events that affect a parent (e.g., the COVID-19 pandemic and its effects on parental PND risk) may also have a pathological effect on their children’s psychological well-being primarily through the poor quality of the affective environment that a parent with psychopathological problems provides to their children [62,63]. 

Specifically, extensive literature has suggested that the relationship between maternal and paternal PND and children’s developmental outcomes is not simply direct, but that the quality of the parent–infant relationship may play a crucial mediation role [64]. Parental–infant bonding is a process of an emotional tie between a mother and/or a father and her/his baby, guided by the caregiver [65], which can be considered as an affective and cognitive dimension of the parent–child relationship [66]. Poor quality of parent–infant bonding is significantly associated with poor quality of parent–infant interactions [67], which in turn is considered a crucial risk factor for infant emotional and behavioral development [68,69]. Several studies conducted before the pandemic have reported significant associations between both maternal and paternal PND and lower quality of parent–infant relationships [70,71,72], evidencing poor parental responsiveness and sensitivity to the child, a predominantly negative affective tone with the baby, and a lower sense of parental competence [73,74,75,76]. 

Overall, previous studies have underlined that the transition to parenthood may represent a critical window of risk for mothers, fathers, and their developing children associated with an increased risk of developing parental and children’s mental problems and a poor quality of parent–child relationships. In this complex scenario, the COVID-19 pandemic and its related restrictions are posing a worrying additional risk, further exacerbating these processes. However, although there have been various systematic reviews investigating the prevalence of maternal and paternal PND during the pandemic (see, for example, [36,37,44,77]), to our best knowledge, there has been no systematic review to date specifically focused on the effect of maternal and paternal PND on the parent–infant relationship and children’s developmental outcomes in the time of COVID-19. This review aimed to fill this literature gap. 

## 2. Materials and Methods

The Guidelines for Accurate and Transparent Health Estimates Reporting (GATHER) [78] (for GATHER checklist, see Appendix A) and the Preferred Reporting Items for Systematic Reviews and Meta-Analyses (PRISMA) guidelines were strictly adhered to in this systematic review [79].

### 2.1. Eligibility Criteria

We included studies that met the following criteria:

∎ Studies evaluating the exposure of maternal or paternal PND on the quality of early parent–infant relationships and/or on infant/toddler outcomes during the COVID-19 pandemic that include or do not include specific measures for assessing COVID-19-related experiences;
∎ Studies including, or comparing, women who gave birth prior to and during the COVID-19 pandemic;
∎ Cohort longitudinal studies in which at least one assessment of PND occurs within the first year postpartum and cross-sectional studies in which the assessment of PND occurs in a period that includes the first postpartum year; in the case of longitudinal studies involving assessment in pregnancy and postpartum, we considered only results related to postpartum time;
∎ Studies reporting outcomes of infants/toddlers up to 36 months;
∎ Studies using acceptable measures of maternal and paternal PND (including self-report scales, clinician rating scales, and structured interviews) of different aspects of the dyadic relationship and infant/toddler outcomes;
∎ Conference or meeting abstracts, provided they met inclusion criteria;
∎ Full-length, peer-reviewed, observational studies including cohort, case-control, and cross-sectional designs, published in English.

The following exclusion criteria applied:

∎ Studies including parents who sought or were in treatment for PND;
∎ Studies including parents with or without a suspected COVID-19 diagnosis;
∎ Studies where PND could not be distinguished from other measures (e.g., ‘psychological distress’, a composite variable combining maternal anxiety and depression);
∎ Studies that did not directly and separately evaluate the effect of maternal and paternal PND on the early dyadic relationship and infant/toddler outcomes (e.g., reported on the combined effect of maternal and paternal postnatal PND and explored the effect of maternal or paternal PND on the dyadic relationship and infant/toddler outcomes as moderator or mediator of other variables);
∎ Studies reporting on the neurodevelopmental outcome of infants/toddlers with a mean or median age of more than 36 months;
∎ Studies conducted among a specific subset of infants not representative of the general population and with a potentially higher risk of developmental delay (e.g., studies including preterm infants, infants with low birth weight, stunted infants, or infants infected with HIV);
∎ Meta-analyses, systematic reviews, nonsystematic reviews, commentaries (included only for reference checks), case studies, and randomized controlled trials.

### 2.2. Information Sources, Search Strategy, and Study Selection

The following seven databases, accessed through “Sapienza” (University of Rome) and Alma Mater Studiorium (University of Bologna) library services, were searched: Medline, CINAHL, SCOPUS, PsycINFO, PsycARTICLES, ScienceDirect, and Web of Science. The search covered the period from 1 December 2019 to 31 July 2022. Pearling was conducted to source relevant publications not indexed in the searched databases. 

The aim was to perform a wide search using keywords for identifying any study of interest conducted during the COVID-19 pandemic related to the impact of maternal and/or paternal PND on different aspects of early dyadic relationships and/or on infant/toddler developmental outcomes. The search was performed according to the searching tools of each database using the following keywords: (1) COVID-19, 2019 novel coronavirus disease, 2019-nCoV disease, SARS-CoV-2, pandemic, coronavirus disease 2019, severe acute respiratory syndrome coronavirus 2, Wuhan coronavirus, 2019-nCoV, novel coronavirus, Wuhan coronavirus, lockdown, quarantine, outbreak; (2) maternal, mother, woman; paternal, father, man, parent; (3) perinatal, peripartum, puerperium, postnatal, postpartum, post-birth, after birth; (4) depression, depressive symptom, depressive disorder, affective disorder, mood disorder, maternity blues; (5) interaction, bond, parenting, relationship, attachment, motherhood, fatherhood, caregiver; (6) neonate, infant, baby, toddler, child, offspring; (7) development, neurodevelopment, motor, language, cognitive, behavior, emotional, social-emotional, social, psychopathology, somatic, delay, risk, outcome, difficulty, function, growth. For the detailed search strategy, see Appendix B.

The search was performed by two independent researchers (E.M. and F.G.) in the allocated databases according to their functions (e.g., Boolean operators such as AND, OR, and NOT, and/or truncation symbols to capture alternative endings and spellings of search terms, as well as filters, namely language: English, publication dates, and ages of infant and toddler: birth–36 months). Each part of the search process was recorded, documented, and cross-checked, and the duplicate extraction was performed via Mendeley software. 

Based on the inclusion and exclusion criteria, the titles, abstracts, and full-text articles retrieved in the initial searches were independently reviewed by two researchers (E.M. and F.G.). The selected articles’ reference sections were also searched, and papers considered potentially relevant were assessed. Studies of all designs were considered, including those whose primary publication was that of a conference or meeting abstract only, provided they met inclusion criteria. The researchers (E.M. and F.G.) compared the eligible studies, and disagreements regarding the inclusion of full-text studies were discussed until consensus was reached. 

### 2.3. Quality Appraisal, Data Extraction, and Analysis

The Joanna Briggs Institute (JBI) Critical Appraisal tools for Cross-Sectional Studies and Cohort Studies were used to assess the methodological quality of the included studies [80]. The tools were adapted, and from each subcategory’s individual rating, an overall score was derived, scoring each study as ‘high’ (meeting >85% of criteria), ‘medium’ (meeting 50–85% of criteria), or ‘low’ (meeting <50% of criteria) quality. The study quality was assessed by two independent researchers (EM and FG) with experience in the critical appraisal of different research designs. Data were extracted and cross-checked independently and captured on a standardized data extraction form. Data were analyzed qualitatively (descriptively) and then synthesized into a narrative synthesis.

### 2.4. Ethical Considerations

This study consists of secondary research; thus, ethical approval was not required for this systematic review.

## 3. Results

### 3.1. Search Results and Overview

**Figure 1:**
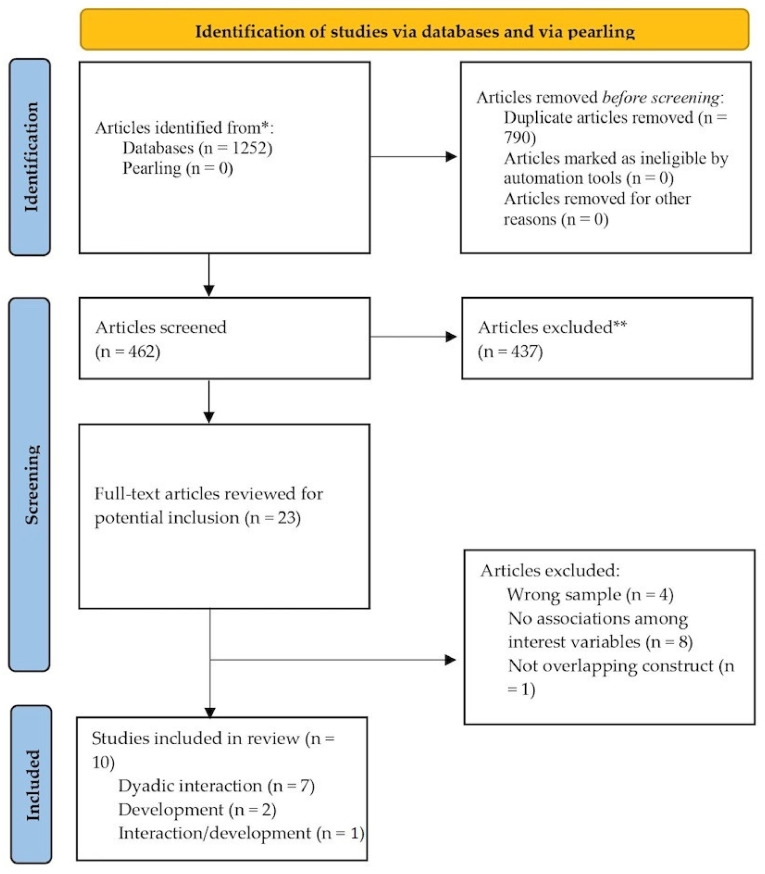
PRIMA flowchart of search results. * Medline, CINAHL, SCOPUS, PsycINFO, PsycARTICLES, ScienceDirect, and Web of Science; ** Based on title and abstract assessment.

The PRISMA flow diagram displays the search strategy and study selection process of this systematic review (Figure 1). A total of 1252 articles were identified in all databases, being 462 of the available number after the exclusion of 790 duplicates. No studies were identified through pearling. Following the title and abstract assessment, 23 articles were selected. After the full-text analyses, 13 studies were excluded according to the eligibility criteria (see Appendix C), and a total of 10 articles were retained for this systematic review. Of the total, 7 articles focused on the impact of postnatal PND on dyadic relationships, 2 focused on the impact of postnatal PND on infant/toddler development, and 1 focused on the impact of postnatal PND on both dyadic relationships and infant/toddler development. For the description of the empirical design of the selected studies and information on the countries where they were conducted, see Table 1 and Table 2, respectively.

### 3.2. Quality of Included Papers

Of the six cross-sectional studies included in this review, four were high-quality [81,82,83,84] and two were medium-quality [85,86]; of the four cohort longitudinal studies, two were high-quality [87,88] and two were medium-quality [89,90]. The most common sources of bias were related to the generic definition of the inclusion/exclusion criteria, the use of self- and parent-reported assessment measures, and the appropriateness of statistical analyses. Most of the studies described in detail the study’s subject and setting, assessed the exposure measured in a valid and reliable way, clearly identified confounding factors as well as strategies to deal with these, and used standard criteria for measurement of the condition. 

**Table 1:** Cross-sectional and cohort longitudinal studies on the impact of PND on early dyadic interaction.

Citation (Country); Study Design	Methodological Quality	Recruitment Periods Related to Pandemic	Sample Size and Characteristics (Recruitment)	Time of Assessment PND; Tools (Referred Scores)	Time of Assessment Interaction; Tools	Results	Control for Confounding
Fernandes et al. [86] *(Portugal)* Cross-sectional	Medium	Prior to pandemic (not specified) April 30–May 21 2020 (lockdown of wave 1)	One group of 567 mothers composed of 414 who gave birth prior to the pandemic and 153 during the pandemic (online)	0–12 months postpartum; HADS (≥11)	0–12 months postpartum; PBQ	Giving birth during pandemic (*p* < 0.001), but not PND levels (*p* = 0.525), predicted lower levels of bonding.	No control for confounding variables.
Oskovi-Kaplan et al. [82] *(Turkey)* Cross-sectional	High	June 2020 (after wave 1)	One group of 233 mothers (maternity ward)	Within 48 h after birth; EPDS (≥13)	Within 48 h after birth; MAI	Depressed mothers showed lower levels of maternal attachment than not depressed ones (*p* < 0.001).	Paired by educational level, maternal age, parity, gestational week of birth, type of delivery, birthweight, and fetal gender.
Liu et al. [85] *(United States)* Cross-sectional	Medium	May 19–August 17 2020 (after wave 1)	One group of 429 mothers (online)	6 months; CES-D (continuous scores)	6 months; MPAS	Higher levels of PND, and concerns regarding COVID-19 pandemic, were associated with lower levels of maternal–infant attachment (*p* < 0.001).	Higher infant age, level of education, and household income were associated with lower levels of attachment, while first pregnancies with higher levels. Maternal age, multiparity, NICU admission, and pandemic duration were not significant.
Erten et al. [81] *(Turkey)* Cross-sectional	High	April–August, 2021 (after wave 2)	One group of 178 mothers (maternity ward)	6 weeks; EPDS (≥13)	6 weeks; MIBQ	Depressed mothers and not depressed ones showed similar levels of bonding (*p* = 0.287).	Paired by education level, maternal age, BMI, previous pregnancy, type of delivery, previous operation history, economic status, employment status, and pregnancy follow-up information; not paired by receiving guest at home during the pandemic (higher for depressed mothers).
Lin et al. [83] *(United States)* Cross-sectional	High	May 19–August 17, 2020 (after wave 1)	One group of 310 women (online)	0–24 months postpartum; CES-D (continuous scores)	0–24 months postpartum; PSI	PND levels contributed to parenting stress, along with concerns regarding the COVID-19 pandemic.	Infant age, number of older children, cohabiting relationships contributed to parenting stress. Infant sex, maternal age, first pregnancy, multiparity, NICU admission, prematurity, maternal race, education, household income, pandemic duration, and anxiety symptoms did not.
Viaux-Savelon et al. [89] *(France)* Cohort longitudinal	Medium	March 27–May 5, 2020 (lockdown of wave 1)	One group of 164 mothers (T1), reduced to 138 (T2) (maternity ward)	10 days (T1) and 6–8 weeks (T2) postpartum; EPDS (<12)	10 days (T1) and 6–8 weeks (T2) postpartum; MIBS	Higher levels of PND at T1 (*p* < 0.001), but not at T2 (*p* = 0.32), were associated with lower level of bonding at T1 and at T2.	Maternal hypertension/preeclampsia, emergency cesarean section, neonatal complications, and threatened preterm labor were associated with higher postnatal PND symptoms.
Handelzalts et al. [87] *(Israel)* Cohort longitudinal	High	February 2018–December 2019 (T1) April 2020–January 2021 (T2) (between wave 1 and 2)	One group of 140 mothers (maternity ward)	6 (T1) and 21 (T2) months postpartum; EPDS (continuous scores)	6 (T1) and 21 (T2) months postpartum; PBQ	Higher levels of PND at T1 and T2 were associated with lower levels of bonding at T1 (*p* < 0.001) and at T2 (*p* < 0.001). At T2, the association between PND and bonding becomes stronger as the concern regarding the COVID-19 pandemic increases.	Age, being primiparous, higher education, having higher income level, lockdown at the time of the survey, past abortions, infertility treatments, epidural and oxytocin administration, and type of delivery were not associated with levels of bonding.
Harrison et al. [90] *(United States)* Cohort longitudinal	Medium	June 2020–February 2021 (after wave 1 and across wave 2)	One group of 125 mothers (pediatric ward)	3 (T1) and 6 (T2) months postpartum; EPDS (continuous scores)	3 (T1) and 6 (T2) months postpartum; items on the frequency of mother–child caretaking activities	Higher levels of PND at T1 predicted fewer recurrent mother–child caretaking activities at T2, even controlling for COVID-19 family impact.	Information not available

HADS: Hamilton Anxiety and Depression Scale; PBQ: Postpartum Bonding Questionnaire; EPDS: Edinburg Postnatal Depression Scale; MAI: Maternal Attachment Inventory; CES-D: Center for Epidemiologic Studies Depression Scale; MPAS: Maternal Postnatal Attachment Scale; MIBQ: Mother-Infant Bonding Questionnaire; PSI: Parental Stress Index; MIBS: Mother-to-Infant Bonding Scale.

**Table 2:** Cross-sectional and longitudinal studies on the impact of PND on infant development.

Citation (Country); Study Design	Methodological Quality	Recruitment Periods Related to Pandemic	Sample Size and Characteristics (recruitment)	Time of Assessment PND; Tools (Referred Scores)	Time of Assessment Infant Outcome; Tools	Results	Control for Confounding
Papadopoulos et al. [88] *(Canada)* Cohort longitudinal	High	May 2020–March 2021 (after wave 2)	One group of 117 mothers (online)	0–2 months postpartum; EPDS (≥14)	0–2 postpartum; the Gross and Fine Motor Scales from the interRAI 0–3 Developmental Domains questionnaire	PND was a significant negative predictor of infant motor outcome (*p* < 0.05).	Country of residence, the month of survey completion, age, and SES were not associated with neonatal motor outcome.
Perez et al. [84] *(Austria)* Cross-sectional	High	Early 2016–early 2019 (for the control cohort) October 2020–November 2020 (for the COVID-19 cohort; wave 2)	One group of 162 mothers composed of 97 recruited prior to the pandemic and 65 during the pandemic (maternity ward)	6–7 months postpartum; EPDS (≥13)	6–7 months postpartum; SFS	PND had a positive, small- to medium-sized effect on sleeping/crying infant regulatory problems (*p* < 0.01). Moderation analysis did not show a significant relevance of being in the COVID-19 or control cohort for the association between depressive symptoms and infant crying/sleeping problems (*p* = 0.383). Regarding infant eating/feeding regulatory problems, there was no significant direct (*p* = 0.215) and interactive effect (*p* = 0.489) of PND.	Women with more previous children reported fewer infant crying/sleeping regulatory problems, whereas the association with infant eating/feeding problems was not significant. Infant negative emotionality was significantly associated with both infant crying/sleeping and eating/feeding problems, whereas maternal perceived social support was not.
Harrison et al. [90] *(United States)* Longitudinal	Medium	June 2020–February 2021 (after wave 1 and across wave 2)	One group of 125 mothers (pediatric ward)	3 (T1) and 6 (T2) months postpartum; EPDS (continuous scores)	3 (T1) and 6 (T2) months postpartum; ASQ-SE-2	PND at 3 months significantly predicted poorer levels of infant social-emotional development at 6 months of life (*p* < 0.05), even after controlling for COVID-19 family impact.	Information not available.

EPDS: Edinburg Postnatal Depressive Scale; SFS: Schreien, Füttern, Schlafen (Questionnaire for Crying, Feeding, and Sleeping); ASQ-SE-2: Ages and Stages Questionnaire-Socioemotional-2.

### 3.3. Association between Maternal and Paternal Postpartum Depression and Early Dyadic Relationships

As previously reported, 8 of the 10 studies included in this review explored the association between PND and early dyadic relationships. All of these studies focused on mothers, neglecting fathers, with samples ranging from 125 [90] to 567 mothers [86]. The recruitment took place at maternity wards (*n* = 4), at pediatric wards (*n* = 1), and online (*n* = 3). Four studies [81,82,87,89] included only low-risk pregnancies, three studies did not exclude potential high-risk pregnancies [83,85,86], and one study did not provide this information [90]. The assessment of PND was conducted with psychometrically validated self-reporting, and the most common tool used was the Edinburg Postnatal Depression Scale (EPDS) [91] (*n* = 5) (see Table 1 for a description of all tools used for PND assessment). Cut-off points, when used, were determined according to the studied population and chosen instrument, but only in two studies were the measures validated for their specific populations [81,86]. The assessment of early dyadic relationships was conducted with psychometrically validated parent-reporting, and the most common tools used were the Postpartum Bonding Questionnaire (PBQ) [92,93] (*n* = 2) and the Mother-to-Infant Bonding Scale (MIBS) [94,95] (*n* = 2) (see Table 1 for a description of all tools used for dyadic relationship assessment). Only two studies reported that the outcome measures were validated for their specific populations [81,86]. Most of the assessments were conducted by call interview (*n* = 3) and online (*n* = 4), and in only one case at a maternity ward (*n* = 1).

#### 3.3.1. Association between Maternal Postpartum Depression and Maternal Bonding/Attachment to the Infant

Six of the eight studies evaluated the association between maternal PND and maternal bonding/attachment to the infant. Among cross-sectional studies, during the first lockdown (wave 1), Fernandes et al. [86] explored the exposure of postnatal PND on the outcome measure within a period between the birth of the baby and the end of the first year postpartum, finding that giving birth during the pandemic, but not PND levels, predicted lower levels of bonding. Conversely, immediately after the first wave of the pandemic, Oskovi-Kaplan et al. [82] found that at two days after birth, depressed mothers showed significantly lower levels of maternal–infant attachment than non-depressed ones. In the same pandemic period, Liu et al. [85] reported that at 6 months postpartum, lower levels of maternal–infant attachment were predicted by higher levels of PND and concerns related to COVID-19 experiences, but not by pandemic duration. Lastly, in the months following the second wave, at 6 weeks after birth, depressed mothers showed similar levels of bonding when compared to non-depressed ones [81]. 

Among longitudinal studies, Viaux-Savelon et al. [89] showed that during the first lockdown (wave 1), higher levels of PND at 10 days postpartum, but not at 6–8 weeks, were associated with lower levels of bonding in both assessments. Similarly, higher levels of PND at 6 and 21 months postpartum, respectively, assessed prior to and during the pandemic, specifically in a period between the first wave (in which 29% of the sample was assessed for PND and bonding) and the end of the second wave, were associated with lower levels of bonding in both assessments, finding that during the pandemic, these associations became stronger as pandemic concerns increased [87].

#### 3.3.2. Association between Maternal Postpartum Depression and the Quality of the Parenting Experience

One of the eight studies evaluated the association between maternal PND and the quality of parenting experiences. This cross-sectional study [83], conducted in the months immediately after the first wave of the pandemic, explored the exposure of PND on the outcome measure within a period between the birth of the baby and the end of the second year postpartum, finding that depressive symptoms and concerns regarding the COVID-19 pandemic, but not pandemic duration, predicted parenting stress levels.

#### 3.3.3. Association between Maternal Postpartum Depression and Frequency of Mother–Child Caretaking Activities

One of the eight studies evaluated the predictive role of maternal PND on the frequency of mother–child caretaking activities. This cohort longitudinal study [90], conducted in a period between the months following the first wave and the second wave of the pandemic, suggested that higher levels of PND at 3 months postpartum predicted fewer recurrent mother–child caretaking activities at 6 months of infant life, even when controlling for COVID-19 family impact.

### 3.4. Association between Maternal and Paternal Postpartum Depression and Infant or Toddler Neurodevelopment

As evidenced above, three of the ten studies included in this review assessed the possible association between PND and infants’/toddlers’ developmental outcomes. No studies were focused on fathers. The sample range of studies was from 117 [88] to 162 [84]. Mothers were recruited through an online procedure (*n* = 1), maternity wards (*n* = 1), and pediatric wards (*n* = 1). One study [84] included only low-risk pregnancies, one did not exclude potential high-risk pregnancies [88], and one study did not provide this information [90]. As is possible to see in Table 2, PND was assessed through the EPDS [91] in all three studies. Infants’/toddlers’ neurodevelopmental outcomes were assessed with parent-reporting tools that are widely psychometrically validated, including the Gross and Fine Motor Scales from the interRAI 0–3 Developmental Domains questionnaire [96] for the assessment of infant motor functioning; the Questionnaire for Crying, Feeding, and Sleeping (German: Fragebogen zum Schreien, Füttern, Schlafen, SFS) [97] for the assessment of infant regulatory problems; and the Ages and Stages Questionnaire-Socioemotional-2 (ASQ-SE-2) [98] for the assessment of infant socioemotional development.

#### 3.4.1. Association between Maternal Postpartum Depression and Infant Motor Development 

One of the three studies evaluated the predictive role of maternal PND on infant motor ability [88] during the second wave of the COVID-19 pandemic. Maternal postnatal PND and infant motor ability were assessed between 0 and 2 months after birth. Results showed that maternal PND was a significant negative predictor of neonatal motor ability.

#### 3.4.2. Association between Maternal Postpartum Depression and Infant Regulatory Problems

One of the three studies evaluated the predictive role of maternal PND on infant regulatory problems in the context of sleeping/crying and eating/feeding [84]. This cross-sectional study was focused on a group of mothers recruited during the second wave of COVID-19 (the COVID-19 cohort) and a group of mothers recruited before the pandemic (the control cohort). The assessment procedure was conducted 6–7 months after childbirth. Results showed that maternal PND had a small- to medium-sized effect on sleeping/crying infant regulatory problems. However, moderation analysis did not show a significant relevance of being in the COVID-19 or control cohort for the association between depressive symptoms and infant crying/sleeping regulatory problems. Regarding infant eating/feeding regulatory problems, both the direct effect of maternal PND and the interactive effect regarding affiliation with a cohort were not significant. 

#### 3.4.3. Association between Maternal Postpartum Depression and Infant Socio-Emotional Development

Finally, one of the three studies evaluated the predictive role of maternal PND on infant socio-emotional development. This longitudinal study [90] found that, controlling for COVID-19 family impact, maternal PND at 3 months significantly predicted poorer levels of infant social-emotional development at 6 months of infant life. 

## 4. Discussion

The COVID-19 pandemic and its related restrictions have led to important changes in people’s daily lives to limit its rapid spread worldwide, severely affecting the mental health of new mothers and fathers [12,14,25]. Epidemiological studies that systematically assessed the impact of COVID-19 on parents during the postnatal period have underlined a dramatic increase in the rates of prevalence of PND among mothers [36,37] and fathers [43,44]. In this scenario, given the extensive evidence accumulated in the pre-pandemic era on the short- and long-term pathogenic effects of parental PND on children’s mental health, one priority challenge has been to explore the possible consequences of maternal and paternal PND on parent–infant relationships and infant development. Based on these premises, this review is the first to explore the possible implications of parental PND during the COVID-19 pandemic on the quality of mother–infant and father–infant relationships and infant developmental outcomes. 

### 4.1. The Effect of Maternal PND during the COVID-19 Pandemic on the Mother–Infant Relationship

The results of this systematic review provide further evidence of a significant association between postpartum depressive symptoms and the quality of early dyadic relationships. 

Notably, six of the eight studies on the impact of PND on different dimensions of the quality of early dyadic relationships reported that across the perinatal period, higher levels of maternal postpartum depression predicted, or were associated with, lower levels of attachment/bonding [82,85,87,89], higher levels of stress related to parenting experiences [83], and a lower frequency of caretaking activities [90]. These associations were found to be significant across the different periods of the pandemic, but they seemed to become stronger as the concern regarding COVID-19 experiences increased [83,85,87]. 

In line with clinical and empirical literature, these results showed that mothers with depressive symptoms may face difficulties in developing affectionate and emotional connections to their infants with a limited range of positive emotions [99,100]. In addition, they may show low tolerance and heightened arousal in response to distress signals from their newborns and the general demands of parenting [101,102], increasing stress related to the perception of not measuring up to their expectations of being parents. The lack of “maternal feeling” along with the “low tolerance to distress” may be attributed to depressive symptoms such as anhedonia, irritability, and low or dysregulated affect, hindering the care of the infants despite the physical and emotional challenges involved in caregiving [103]. 

In addition, this systematic investigation seems to confirm that new mothers, in addition to representing a specific, vulnerable population by themselves, are particularly at risk of developing mental disorders during health or social disasters [15], with consequences for parenting and child development [104]. The COVID-19 pandemic has profoundly shaken the daily life of people, increasing social isolation, care restriction, and psychic suffering, and it has generated a wave of fear in mothers (e.g., fear about the effect of the virus on the fetus and the newborn, fear of attending medical visits to avoid the risk contamination, fear of the effect of stress on the fetus’ and baby’s growth, and fear of vaccination), adding further burden to the intrapsychic and interpersonal challenges involved in the process of the transition to parenthood [105]. 

It is worth noting that mothers included in these studies are mainly representative of low-risk populations, explaining at least in part why two of the eight studies included in this systematic review did not find any impact of PND on early dyadic relationships. It is possible, in fact, that given the reorganization of family life determined by the pandemic and policies to fight the virus, low-risk mothers might have experienced an opportunity to forge a new family cohesion, develop strong triadic relationships, deepen interpersonal relationships, and develop new partnership skills within couples, factors known to be protective for perinatal mental health and its consequences [106,107]. In line with previous empirical findings [108,109], this consideration implies that in high-risk contexts (such as economic precariousness, social isolation, recent immigration, single motherhood, history of psychiatric illness, etc.), the detrimental impact of maternal postpartum depression, particularly in cases of extraordinary events, could worsen.

From a clinical perspective, these results suggest that healthcare professionals must channel increased energy into addressing maternal depression and its effects, above all during states of emergency, offering a response to the specific need of this population [110]. 

Particularly, they suggest that it is crucial to prevent the appearance of mental disorders before they disrupt mothers’ behavior through the implementation of early assessment programs and follow-up. These strategies should involve collaboration among midwives, obstetric care, maternal wards, pediatricians, and perinatal psychiatric care to avoid losing sight of suffering women, especially those at higher risk who often experience difficulty in seeking help from professionals. Moreover, they suggest that it is essential to implement specific perinatal psychiatric care programs for cases at risk for clinical depression. These programs should include therapeutic interventions for reducing symptomatology, sustaining parenting, and limiting the consequences for infant development, and therefore the entire family functioning, as well as follow-up assessments for monitoring the course of symptoms [106]. Beyond these considerations, given the risk of a temporary suspension or reduction of care visits during emergencies, the need has emerged for setting up and integrating new care systems to maintain continuity of care and contact with mothers [111]. In this sense, the use of digital platforms might help to implement early screening strategies to identify mothers at risk and to propose, according to each specific case, early, graduated intervention [112]. 

### 4.2. The Effect of Maternal PND on Infant Development during the COVID-19 Pandemic

There were very few results regarding maternal PND on children’s (aged 0 to 3 years) developmental outcomes. We included in the present systematic review only three studies that showed a significant impact of maternal PND on infant motor development [88], infant self-regulation abilities [84], and infant socio-emotional development [90]. One longitudinal study was conducted from after the so-called first wave of the COVID-19 pandemic and across the second wave [90], whereas the other two studies explored the effect of maternal PND on infant developmental outcomes during the second wave [84,88]. 

Specifically, in line with previous studies conducted before the pandemic [46,113,114], the study by Papadopoulos et al. [88] evidenced that maternal PND significantly predicted difficulties in infant motor ability during the first 2 months of life. A possible explanation can be that maternal PND significantly affects the quality of early mother–infant relationships [70,71,72]. Indeed, extensive literature has shown that in the presence of maternal PND, mother–infant interactions are characterized by low maternal sensitivity, responsiveness, and contingency to the child’s needs [73,74,75]. Consequently, these relational patterns would lead to the child experiencing greater discomfort and less security within dyadic interactional exchanges, resulting in poor engagement in motor exploration [115]. As the results of our systematic review have also demonstrated, the COVID-19 pandemic has further exacerbated these processes, leading to a lower quality of mother–infant relationships among mothers with PND (i.e., poorer quality of maternal bonding to infants, higher parenting stress levels, and fewer recurrent mother–child caretaking activities) that, in turn, may affect infant motor outcomes. Further research is needed to also confirm these links during childhood. 

Moreover, the study by Perez et al. [84] showed that maternal PND significantly predicted infant regulatory problems in the context of sleeping/crying at 6–7 months after childbirth, but not in the context of eating/feeding problems. However, although mothers in the COVID-19 cohort and those with greater PND had infants with more sleeping/crying regulatory problems compared to mothers in the control group, being in the COVID-19 group was not a significant predictor of infant outcomes. Overall, this study has confirmed the association between maternal PND and infant regulatory problems [116,117]. However, the effect of the pandemic on infant outcomes was independent of those exerted by maternal depressive symptoms, suggesting that other potential mediators may explain the complex relationship between maternal PND during the pandemic and infant regulatory behaviors. Particularly, infant self-regulation during the first year of life is closely associated with the mother’s ability to co-regulate within the context of reciprocal dyadic interactions [118]. Consequently, the poor quality of mother–infant interaction found among mothers with PND both before [70,71,72] that since the beginning of the COVID-19 pandemic [81,82,83,86,87,90,111] may further increase the risk of infant regulatory problems. For example, the longitudinal study by Provenzi et al. [119] conducted during the pandemic on a sample of mothers with postnatal anxiety found that maternal parenting stress significantly mediated the effect of maternal anxiety during the postpartum period and infant regulatory problems at 3 months of age. To date, no study has yet explored the possible mediation role played by the quality of affective environment provided by depressed postnatal mothers to their children on infant regulatory outcomes, and future research should explore this direction to fill this important gap. 

Finally, the longitudinal study by Harrison et al. [90] found that maternal PND at 3 months significantly predicted poorer levels of infant social-emotional development at 6 months of life. Significant associations between maternal PND and child social-emotional functioning have been shown in several studies conducted before the pandemic [49,120]. Again, one possible pathway of risk transmission between maternal PND and infant socio-emotional problems has been suggested to be through the impact of maternal depressive symptoms on the quality of the mother–infant relationship [121]. Consequently, although the study by Harrison et al. [90] provided only preliminary results on the effect of maternal PND during the COVID-19 pandemic on infant socio-emotional problems, as evidenced above, it also found that maternal PND was significantly associated with fewer concurrent mother–child caretaking activities. 

In light of these findings, it is important to note that studies included in this review were focused on high socioeconomic-status groups, and excluded infants with complications at birth or in the postpartum period, variables that the literature has shown to further exacerbate the psychological impact of COVID-19 on families’ mental health [122]. 

### 4.3. The Role of Paternal PND during the COVID-19 Pandemic

Recent evidence has underlined that new fathers are a population group at higher risk for the onset of psychopathology, especially PND [10,38,39]. However, compared to new mothers, fathers have received little attention, even before the COVID-19 outbreak, and only a few studies have explored the impact of the pandemic on the rate of prevalence of paternal PND [43,44,45]. The results of our systematic review on the possible effect of maternal and paternal PND on the quality of parent–infant relationships and infant developmental outcomes have further confirmed this trend. Indeed, we found no study specifically focused on the relationship between paternal PND and the outcomes of interest of this study. Nevertheless, emerging pre-pandemic research has underlined the key role played by paternal PND, both on the quality of relationships with children [13] and on children’s developmental outcomes [123]. For example, the study by Musser et al. [41] evidenced a significant association between paternal PND and a low quality of infant bonding, that in turn was associated with a wide range of infant developmental difficulties (e.g., emotional problems, anxiety/depressive symptoms, and behavioral problems). Other studies have shown that depressed fathers are less engaged with their infants [13], manifest more withdrawal, and show decreased stimulation during father–infant interactions [124,125]. Interestingly, paternal PND has been suggested to be an additional risk factor for the onset and maintenance of maternal PND [126]. Notably, paternal psychopathological problems also negatively impacted their wives’ supportive relationships with children [127] and the related consequences for newborns [44]. Overall, given the growing evidence of the crucial role played by fathers’ mental health during the postpartum period on the family’s psychological well-being, it is essential to increase research on the possible consequences that paternal PND during the pandemic may have had on fathers’ relationships with their children and their development. Future findings in this area can be used to implement prevention and intervention programs to mitigate the short- and long-term effects of paternal PND on children’s well-being. 

## 5. Limitations and Future Directions

The findings of this review have to be seen in the light of some limitations. First, given the paucity of studies on the topic, this review includes a limited amount of study, and the evidence that emerged should be treated with caution. 

Additionally, only one study [86] included a group of mothers composed of both mothers who gave birth before the pandemic and during the pandemic, exploring the specific role of giving birth during the pandemic on the associations between PND and early dyadic relations; only one study [87] longitudinally compared a group of mothers, exploring the association between PND and early dyadic relations assessed prior to and during the pandemic; and only one study [84] examined the influence of PND on infant regulatory problems, comparing mothers recruited before and during the pandemic. For this reason, it is difficult to point out the differences between the effects of PND during a pandemic and in another period. Moreover, only three studies [83,85,87] specifically explored the extent of the contribution of concerns related to the pandemic on the interested associations. Therefore, it is not possible to draw conclusions about the extent of the impact of the pandemic, and future studies are needed. Finally, we included in this systematic review the study by Harrison et al. [90], which, to date, is only available as a published meeting abstract. However, although we are conscious that the conference/meeting abstracts may contain only partial information, we have chosen to include them in this systematic review both for the relevant findings reported and for the poverty of studies in the field, as suggested by a recent systematic review by Scherer and Saldanha [128] on the utility of including meeting abstracts in a systematic review.

Essentially, all of the studies included in this review resorted to self-reporting and report-form tools to assess PND, early dyadic relationships, and infants’ development. Even though these measures are commonly administered in studies addressing maternal psychological functioning and its effects, and their massive use is justified by the pandemic restrictions, biased responses may not be excluded. In addition, in half of the studies, the sample was collected online, which could lead to self-selection bias since mothers who participated were likely to be more motivated and interested in the subject than those of the general population. In line with this consideration, most of the studies were conducted in low-risk groups of mothers. Therefore, these findings cannot be generalized, and their clinical implication should take into account consideration the characteristics of the sample. 

Moreover, even if most of the studies included in this review considered additional variables (i.e., social support, socio-economic status, parity, educational level, etc.) that may buffer the impact of PND on interested variables during the COVID-19 pandemic (e.g., [129]), future studies should investigate the interrelationship between these variables through path analysis and linear structural relation modeling to understand their contributions to the outcomes for mothers, dyadic relationships, and infants.

Lastly, given the lack of empirical studies on fathers, the increase in clinical and empirical interest in fathers and in broader family relationships to better understand the consequences of maternal PND is mandatory.

## 6. Conclusions

This systematic review provides an overview of the most recent advances on the impact of maternal and paternal PND on the quality of dyadic relationships and child development in the first three years of life during the COVID-19 pandemic. Findings on mothers confirmed a detrimental effect of PND both on the quality of dyadic relationships and child outcomes. They also suggested that PND consequences might be worse in cases of stressful and traumatic events, such that of the COVID-19 outbreak. Findings on fathers are missing. 

Overall, the findings of this systematic review add new evidence in support of the life-course perspective [58,59]. Specifically, in accordance with the notion of “linked lives” [60], our results confirmed that the presence of parental PND represents a crucial risk factor for children’s emotional–behavioral development, particularly through its effects on the affective environment that parents with PND provide to their children (i.e., a poor quality of early parent–infant relationships). Notably, the COVID-19 pandemic has been confirmed to be an additional risk factor in these processes. Further studies should explore possible long-term consequences for childhood development that may persist across the lifespan. 

These findings suggest the need to prevent the occurrence of PND through early assessment programs and follow-up, also fostering interdisciplinary collaborations among professionals working in the perinatal field. Additionally, they suggest the need to implement therapeutic interventions for preventing the short- and long-term consequences of PND at individual and dyadic. They also underline the need for setting up or integrating new care systems (e.g., the use of digital platforms) to maintain continuity of care in cases of emergencies. Lastly, they further indicate the urgency of including fathers in the practices of perinatal health services as well as in research programs.

## Data Availability

No new data were created or analyzed in this study. Data sharing is not applicable to this article.

## Appendix A

**Table A1:** GATHER checklist.

Item #	Checklist Item	Reported on Page #
**Objectives and funding**		
**1**	Define the indicator(s), populations (including age, sex, and geographic entities), and time period(s) for which estimates were made.	Introduction pg. 1–3
**2**	List the funding sources for the work.	Funding pg. 18
**Data Inputs**		
*For all data inputs from multiple sources that are synthesized as part of the study:*		
**3**	Describe how the data were identified and how the data were accessed.	Materials and Methods pg. 4–6; Appendix A
**4**	Specify the inclusion and exclusion criteria. Identify all ad-hoc exclusions.	Materials and Methods pg. 3–4; Appendix B
**5**	Provide information on all included data sources and their main characteristics. For each data source used, report reference information or contact name/institution, population represented, data collection method, year(s) of data collection, sex and age range, diagnostic criteria or measurement method, and sample size, as relevant.	Results pg. 6–7; Table 1; Results pg. 10–11; Table 2
**6**	Identify and describe any categories of input data that have potentially important biases (e.g., based on characteristics listed in item 5).	Results pg. 6–7; Results pg. 10–11; Materials and Methods pg. 5; Results pg. 6
*For data inputs that contribute to the analysis but were not synthesized as part of the study:*		
**7**	Describe and give sources for any other data inputs.	Appendix A
*For all data inputs:*		
**8**	Provide all data inputs in a file format from which data can be efficiently extracted (e.g., a spreadsheet rather than a PDF), including all relevant meta-data listed in item 5. For any data inputs that cannot be shared because of ethical or legal reasons, such as third-party ownership, provide a contact name or the name of the institution that retains the right to the data.	Appendix C
**Data analysis**		
**9**	Provide a conceptual overview of the data analysis method. A diagram may be helpful.	Materials and Methods pg. 4–5; Appendix A
**10**	Provide a detailed description of all steps of the analysis, including mathematical formulae. This description should cover, as relevant, data cleaning, data pre-processing, data adjustments and weighting of data sources, and mathematical or statistical model(s).	Materials and Methods pg. 4–5; Appendix A
**11**	Describe how candidate models were evaluated and how the final model(s) were selected.	N/A
**12**	Provide the results of an evaluation of model performance, if performed, as well as the results of any relevant sensitivity analysis.	N/A
**13**	Describe methods for calculating uncertainty of the estimates. State which sources of uncertainty were and were not accounted for in the uncertainty analysis.	Materials and Methods pg. 5–6
**14**	State how analytic or statistical source code used to generate estimates can be accessed.	N/A
**Results and Discussion**		
**15**	Provide published estimates in a file format from which data can be efficiently extracted.	Table 1 and Table 2
**16**	Report a quantitative measure of the uncertainty of the estimates (e.g., uncertainty intervals).	Table 1 and Table 2
**17**	Interpret results in light of existing evidence. If updating a previous set of estimates, describe the reasons for changes in estimates.	Discussion pg. 13
**18**	Discuss limitations of the estimates. Include a discussion of any modelling assumptions or data limitations that affect interpretation of the estimates.	Discussion pg. 13; Limitations and Future Directions pg. 16–17; Conclusions pg. 17

## Appendix B

**Table A2:** Tabulated search terms for each database.

**Search strategy for the impact of maternal and/or paternal postpartum depression on the quality of early reltionships** (“COVID-19” OR “COVID19” OR “2019 novel coronavirus disease” OR “2019-nCoV disease” OR “SARS-CoV-2” OR pandemic OR “coronavirus disease 2019” OR “severe acute respiratory syndrome coronavirus 2” OR “Wuhan coronavirus” OR “2019-nCoV” OR “novel coronavirus” OR “Wuhan coronavirus” OR lockdown OR quarantine OR outbreak) AND ((maternal OR mother* OR wom*n) OR (paternal OR father* OR man) OR (parent*)) AND ((perinatal OR peripartum OR puerper* OR postnatal OR postpartum OR post-birth OR “after birth”) AND (depress* OR “depress* symptom*” OR “depressive disorder*” OR “affective disorder*” OR “mood disorder*” OR “maternity blues”)) AND (interaction* OR bond* OR parenting OR relationship* OR attachment OR motherhood OR fatherhood OR caregiv*) **Search strategy for the impact of maternal and/or paternal postpartum depression on infant/toddler outcomes** (“COVID-19” OR “COVID19” OR “2019 novel coronavirus disease” OR “2019-nCoV disease” OR “SARS-CoV-2” OR pandemic OR “coronavirus disease 2019” OR “severe acute respiratory syndrome coronavirus 2” OR “Wuhan coronavirus” OR “2019-nCoV” or “novel coronavirus” OR “Wuhan coronavirus” OR lockdown OR quarantine OR outbreak) AND ((maternal OR mother* OR wom*n) OR (paternal OR father* OR man) OR (parent*)) AND ((perinatal OR peripartum OR puerper* OR postnatal OR postpartum OR post-birth OR “after birth”) AND (depress* OR “depress* symptom*” OR “depressive disorder*” OR “affective disorder*” OR “mood disorder*” OR “maternity blues”)) AND (neonate* OR infant* OR bab* OR toddler OR child* OR offspring*) AND (development* OR neurodevelopment OR motor OR language OR cognitive OR behavior OR emotional OR social-emotional OR social OR psychopatholog* OR somatic OR delay* OR risk* OR outcome* OR difficult* OR function* or growth) **Filters** Age: birth-36 months Publication dates: 2019–2022 Languages: English

## Appendix C

**Table A3:** List of studies excluded at full text review stage and reasons for exclusion.

Excluded Reference		Exclusion Reason
**1**	Chang, O.; Layton, H.; Amani, B.; Merza, D.; Owais, S.; Van Lieshout, R.J. The Impact of the COVID-19 Pandemic on the Mental Health of Women Seeking Treatment for Postpartum Depression. *J. Matern. Fetal Neonatal Med.* **2022**, *35*, 9086–9092. https://doi.org/10.3390/ijerph19137833.	Mothers seeking treatment for PND
**2**	Duguay, G.; Garon-Bissonnette, J.; Lemieux, R.; Dubois-Comtois, K.; Mayrand, K.; Berthelot, N. Socioemotional Development in Infants of Pregnant Women During the COVID-19 Pandemic: The Role of Prenatal and Postnatal Maternal Distress. *Child Adolesc. Psychiatry Ment. Health* **2022**, *16*, 28. https://doi.org/10.1186/s13034-022-00458-x	Assessment of a combined measure of anxious depressive symptoms
**3**	Fernandes, D.V.; Canavarro, M.C.; Moreira, H. Postpartum during COVID-19 Pandemic: Portuguese Mothers’ Mental Health, Mindful Parenting, and Mother–Infant Bonding. *J. Clin. Psych.* **2021**, *77*, 1997–2010. https://doi.org/10.1002/jclp.23130.	No assessment of the impact of PND on dyadic relationships
**4**	Fernandes, D.V.; Canavarro, M.C.; Moreira, H. The Role of Mothers’ Self-Compassion on Mother–Infant Bonding during the COVID-19 Pandemic: A Longitudinal Study Exploring the Mediating Role of Mindful Parenting and Parenting Stress in the Postpartum Period. *Infant Ment Health* **2021**, *42*, 651–635. https://doi.org/10.1002/imhj.21942.	No assessment of the impact of PND on dyadic relationships
**5**	Fernandes, D.V.; Canavarro, M.C.; Moreira, H. Self-Compassion and Mindful Parenting among Postpartum Mothers During The COVID-19 Pandemic: The Role Of Depressive and Anxious Symptoms. *Current Psych.* **2022**. https://doi.org/10.1007/s12144-022-02959-6.	PND as a mediator
**6**	Gonzalez-Garcia, V.; Exertier, M.; Denis, A. Anxiety, Post-Traumatic Stress Symptoms, and Emotion Regulation: A Longitudinal Study of Pregnant Women Having Given Birth during the COVID-19 Pandemic. *Eur. J. Trauma Dissoc.* **2021**, *5*, 1002255. https://doi.org/10.1016/j.ejtd.2021.100225.	No associations between PND and infant/toddler outcomes
**7**	Kornfield, S.L.; White, L.K.; Waller, R.; Njoroge, W.; Barzilay, R.; Chaiyachati, B.H.; Himes, M.M.; Rodriguez, Y.; Riis, V.; Simonette, K.; Elovitz, M.A. Gur, R.E. Risk and Resilience Factors Influencing Postpartum Depression and Mother–Infant Bonding During COVID-19. 2021 *Health Aff.* **2021**, *40*, 1566–1574. https://doi.org/10.1377/hlthaff.2021.00803.	No assessment of the impact of PND on dyadic relationships
**8**	Layton, H.; Owais, S.; Savoy, C.D.; Van Lieshout, R.J. Depression, Anxiety, and Mother–Infant Bonding in Women Seeking Treatment for Postpartum Depression Before and During the COVID-19 Pandemic. *J. Clin. Psych.* **2022**, *82*, 21m13874. https://doi.org/10.4088/jcp.21m13874.	Mothers seeking treatment for PND
**9**	Peng, S.; Yi, Z.; Hongyan, L.; Xiaona, H.; Douglas, J.N.; Lixia, Y.; Wei, L.; Yahiu, L.; Huaping, Z.; Li, C.; Chunhua, L.; Yang, C.; Pei, Z.; Shiwen, X.; Anuradha, N. A Multi-Center Survey on the Postpartum Mental Health of Mothers and Attachment to their Neonates during COVID-19 in Hubei Province of China. *Ann. Transl. Med.* **2021**, *9*, 382. https://doi.org/10.21037/atm-20-6115.	Mothers suspected with COVID-19 compared to mothers without COVID-19
**10**	van den Heuvel, M.I.; Vacaru, S.V.; Boekhorst, M.G.B.M.; Cloin, M.; van Bakel, H.; Riem, M.M.E.; de Weerth, C.; Beijers, R. Parents of Young Infants Report Poor Mental Health and more Insensitive Parenting during the First COVID-19 Lockdown. *BMC Pregnancy Childbirth* **2022**, *22*, 302, https://doi.org/10.1186/s12884-022-04618-x.	No associations between PND and dyadic relationships
**11**	Werchan, D.; Hendrix, C.; Hume, A.M.; Zhang, M.; Thomason, M.E.; Brito, N.H. *Cognitive and Socioemotional Development in Infants Exposed to SARS-CoV-2 in Utero: A Moderating Role of Prenatal Psychosocial Stress on Infant Outcomes*; PsyArXiv, **2022**	Infants exposed to SARS-CoV-2 in utero
**12**	Wu, Y.; Espinosa, K.M.; Barnett, S.D.; Kapse, A.; Quistorff, J.L.; Lopez, C.; Andescavage, N.; Pradhan, S.; Lu, Y.-C.; Kapse, K.; et al. Association of Elevated Maternal Psychological Distress, Altered Fetal Brain, and Offspring Cognitive and Social-Emotional Outcomes at 18 Months. *JAMA Netw. Open* **2022**, *5*, e229244	No assessment of PND after childbirth
**13**	Youji, T.; Naohisa, T.; Yuri, A.; Kazuyo, F.; Momoko, I.; Takashi, U.; Megumu, I.; Yasuo, A.; Masafumi, M.; Takahiro, N. Psychological Impacts of the COVID-19 Pandemic on One-Month Postpartum Mothers in a Metropolitan Area of Japan. *BMC Pregnancy & Childbirth* **2021**, *21*, 845. https://doi.org/10.1186/s12884-021-04331-1	No assessment of the impact of PND on dyadic relationships

## Appendix D

**Table A4:** Key extracted and used data.

Study (Author, Ref)	Population Represented	Data Collection Method	Years of Data Collection	Measurement Method	Sample Size
Erten et al. [80]	Low-risk mothers (no high-risk pregnancies or psychiatric disease)	At maternity ward	April–August, 2021	Self-report for PND Parent-report for dyadic relationships	178
Fernandes et al. [85]	Age ≥ 18 years	Online recruitment	Prior to pandemic (not specified) 30 April –21 May 2020	Self-report for PND Parent-report for dyadic relationships	567
Handelzalts et al. [86]	Singleton birth (no preterm birth)	At maternity ward	February 2018–December 2019 April 2020–January 2021	Self-report for PND Parent-report for dyadic relationships	140
Harrison et al. [89]	N/A	At pediatric ward	June 2020–February 2021	Self-report for PND Parent-report for dyadic relationships and developmental outcome	125
Lin et al. [82]	Age ≥ 18 years	Online recruitment	19 May–17 August 2020	Self-report for PND Parent-report for dyadic relationships	310
Liu et al. [84]	Age ≥ 18 years	Online recruitment	19 May–17 August 2020	Self-report for PND Parent-report for dyadic relationships	429
Oskovi-Kaplan et al. [81]	Low-risk mothers (no high-risk pregnancies, psychiatric diseases, COVID-19 diseases during pregnancy, or a relative with COVID-19 disease)	At maternity ward	June 2020	Self-report for PND Parent-report for dyadic relationships	233
Papadopoulos et al. [87]	Age 18–55 years and singleton or multiple birth	Online recruitment	May 2020–March 2021	Self-report for PND Parent-report for developmental outcome	117
Perez et al. [83]	Age ≥ 18 years and singleton birth (no high-risk pregnancies, substance abuse, premature birth, or low birth weight)	At maternity ward	Early 2016–early 2019 October 2020–November 2020	Self-report for PND Parent-report developmental outcome	162
Viaux-Savelon et al. [88]	Age ≥ 18 years and post-delivery hospitalization in the conventional postnatal ward	At maternity ward	27 March–5 May 2020	Self-report for PND Parent-report for dyadic relationships	164 (T1) 132 (T2)
